# Supplementary material for: Addressing people’s current and future states in a reinforcement learning algorithm for persuading to quit smoking and to be physically active
Source: PLoS One. 2022 Dec 1;17(12):e0277295. doi: 10.1371/journal.pone.0277295 (PMC9714722; doi:10.1371/journal.pone.0277295)
Supplement: S1 Appendix — Table that displays examples of persuasive message templates and resulting messages. (PDF) [file pone.0277295.s001.pdf]

Table that displays examples of persuasive message templates and resulting messages.

**Persuasion Type**	**Template Example**	**Message Example**
Action planning	Please think about the time after this and before the next session. When, where and how do you plan to do your recommended preparation activity? Please formulate a rule for completing your activity that has the form "If ⟨situation⟩, then I will ⟨do activity⟩." For instance, "If I get out of the shower tonight, then I will ⟨*do activity*⟩." Type your rule into this chat. The more precise, concrete and personally you formulate your rule, the more it can help you.	Please think about the time after this and before the next session. When, where and how do you plan to do your recommended preparation activity? Please formulate a rule for completing your activity that has the form "If ⟨situation⟩, then I will ⟨do activity⟩." For instance, "If I get out of the shower tonight, then I will *identify reasons why I want to stop smoking.*" Type your rule into this chat. The more precise, concrete and personally you formulate your rule, the more it can help you.
Authority	Experts recommend ⟨*doing activity*⟩ to ⟨*positive impact of activity*⟩.	Experts recommend *identifying reasons why you want to stop smoking* to *increase your aspiration to stop smoking.*
Commitment	You've committed to become somebody who has quit smoking. ⟨*doing activity*⟩ may help you to become this person.	You've committed to become somebody who has quit smoking. *Identifying reasons why you want to stop smoking* may help you to become this person.
Consensus	Most people think that ⟨*doing activity*⟩ may help to ⟨*positive impact of ac−tivity*⟩.	Most people think that *identifying reasons why you want to stop smoking* may may help to *increase your aspiration to stop smoking.*
