# Supplementary material for: Addressing people’s current and future states in a reinforcement learning algorithm for persuading to quit smoking and to be physically active
Source: PLoS One. 2022 Dec 1;17(12):e0277295. doi: 10.1371/journal.pone.0277295 (PMC9714722; doi:10.1371/journal.pone.0277295)
Supplement: S2 Appendix — Figure that shows the participant flow through the study components in the experiment. The numbers next to the downward arrows denote how many people started the study components. We show the distribution across the four algorithm complexity levels for the participants who did not respond to the invitation to a study component after the randomization. Note that participants can return their submission on Prolific to withdraw from a study. (PDF) [file pone.0277295.s002.pdf]

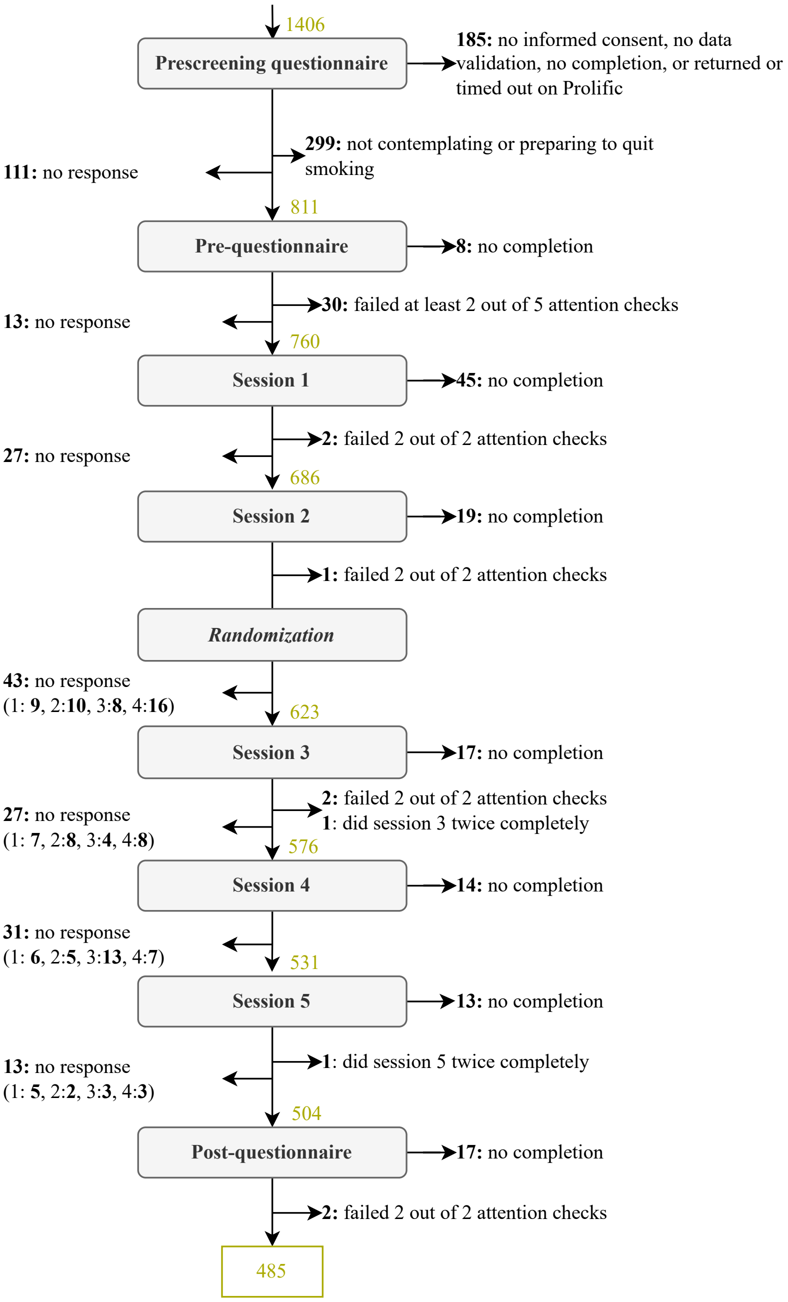

Figure that shows the participant flow through the study components in the experiment. The numbers next to the downward arrows denote how many people started the study components. We show the distribution across the four algorithm complexity levels for the participants who did not respond to the invitation to a study component after the randomization. Note that participants can return their submission on Prolific to withdraw from a study.
