# Supplementary material for: Addressing people’s current and future states in a reinforcement learning algorithm for persuading to quit smoking and to be physically active
Source: PLoS One. 2022 Dec 1;17(12):e0277295. doi: 10.1371/journal.pone.0277295 (PMC9714722; doi:10.1371/journal.pone.0277295)
Supplement: S3 Appendix — Table that depicts the participant characteristics for each algorithm complexity level. (PDF) [file pone.0277295.s003.pdf]

Table that depicts the participant characteristics for each algorithm complexity level.

	**Algorithm Complexity Level**			
**Characteristic**	**1**	**2**	**3**	**4**
Number				
- n	162	167	163	174
Gender				
- Female, n (%)	84 (51.85%)	87 (52.10%)	83 (50.92%)	91 (52.30%)
- Male, n (%)	76 (46.91%)	76 (45.51%)	78 (47.85%)	79 (45.40%)
Age				
- 18–30, n (%)	73 (45.06%)	84 (50.30%)	81 (49.69%)	74 (42.53%)
- 31–40, n (%)	47 (29.01%)	31 (18.56%)	38 (23.31%)	50 (28.74%)
- 41–50, n (%)	21 (12.96%)	29 (17.37%)	19 (11.66%)	31 (17.82%)
- 51–60, n (%)	15 (9.26%)	20 (11.98%)	20 (12.27%)	15 (8.62%)
- 61–74, n (%)	6 (3.70%)	3 (1.80%)	5 (3.07%)	4 (2.30%)
Big-5 Personality				
- Agr., Mean (SD)	4.83 (1.17)	4.90 (1.11)	4.85 (1.15)	4.86 (1.14)
- Con., Mean (SD)	4.89 (1.41)	4.88 (1.42)	4.93 (1.45)	4.94 (1.38)
- ES, Mean (SD)	4.23 (1.60)	4.21 (1.57)	4.15 (1.55)	4.16 (1.60)
- Ext., Mean (SD)	3.90 (1.61)	3.89 (1.59)	3.98 (1.63)	3.93 (1.59)
- OE, Mean (SD)	5.20 (1.15)	5.22 (1.13)	5.23 (1.15)	5.19 (1.14)
Dropout after Session 3*				
- No response, n (%)	18 (11.11%)	15 (8.98%)	20 (12.27%)	18 (10.34%)
Effort for First Activity				
- Mean (SD)	5.55 (2.52)	5.54 (2.54)	5.48 (2.56)	5.45 (2.47)
Previous Quit Attempt of at Least 24 Hours				
- Yes, n (%)	128 (79.01%)	127 (76.05%)	135 (82.82%)	133 (76.44%)
- No, n (%)	34 (20.99%)	40 (23.95%)	28 (17.18%)	41 (23.56%)
TTM-stage for Becoming Physically Active				
- Precontemplation, n (%)	9 (5.56%)	14 (8.38%)	10 (6.13%)	10 (5.75%)
- Contemplation, n (%)	44 (27.16%)	43 (25.75%)	49 (30.06%)	50 (28.74%)
- Preparation, n (%)	43 (26.54%)	41 (24.55%)	38 (23.31%)	37 (21.26%)
- Action, n (%)	19 (11.73%)	19 (11.38%)	19 (11.66%)	25 (14.37%)
- Maintenance, n (%)	47 (29.01%)	50 (29.94%)	47 (28.83%)	52 (29.89%)
TTM-stage for Quitting Smoking				
- Contemplation, n (%)	133 (82.10%)	147 (88.02%)	140 (85.89%)	152 (87.36%)
- Preparation, n (%)	29 (17.90%)	20 (11.98%)	23 (14.11%)	22 (12.64%)

Abbreviations: SD, Standard deviation; Agr., Agreeableness, Con., Conscientiousness; ES, Emotional stability; Ext., Extraversion; OE, Openness to experience.
* Session 3 was the first session in which participants were treated differently based on their condition (i.e., persuaded based on the four algorithm complexity levels).
