# Supplementary material for: Addressing people’s current and future states in a reinforcement learning algorithm for persuading to quit smoking and to be physically active
Source: PLoS One. 2022 Dec 1;17(12):e0277295. doi: 10.1371/journal.pone.0277295 (PMC9714722; doi:10.1371/journal.pone.0277295)
Supplement: S4 Appendix — Figure that presents the structure of the five conversational sessions with the virtual coach Sam. (PDF) [file pone.0277295.s004.pdf]

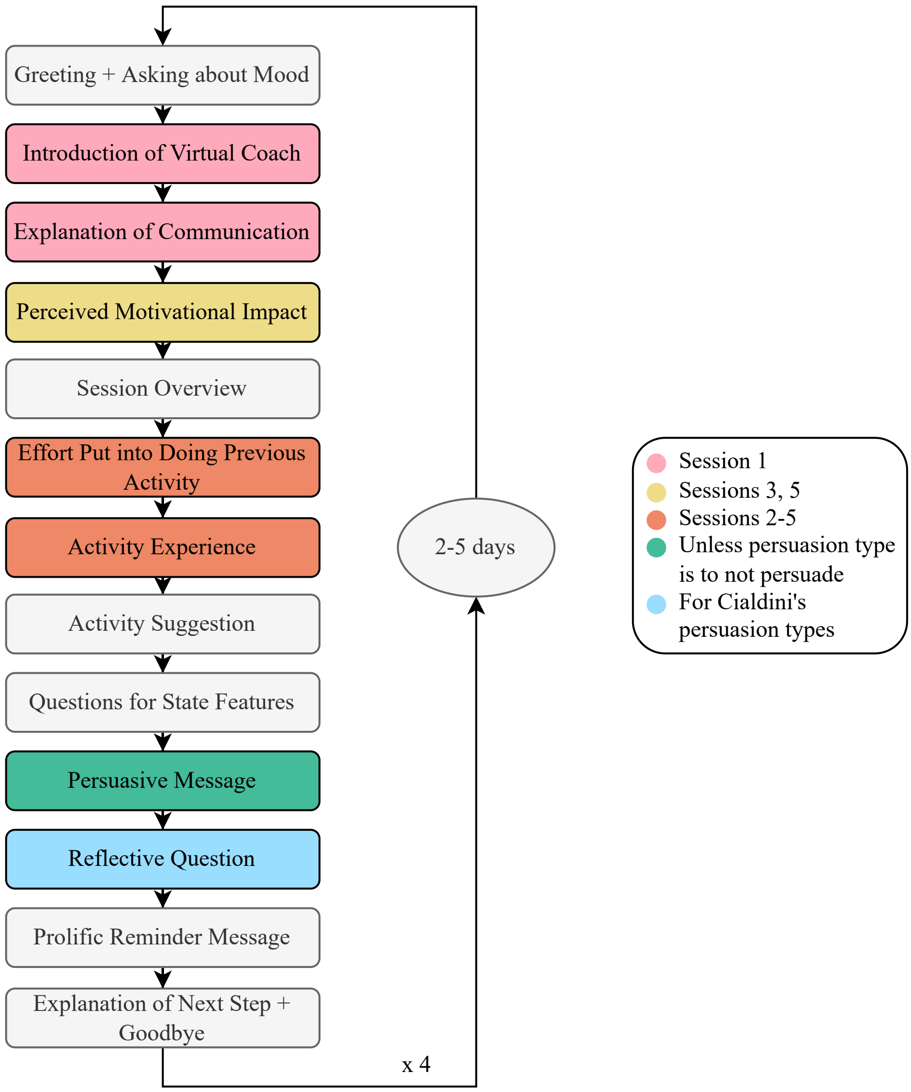

Figure that visualizes the structure of the five conversational sessions with the virtual coach Sam.
