# Supplementary material for: Addressing people’s current and future states in a reinforcement learning algorithm for persuading to quit smoking and to be physically active
Source: PLoS One. 2022 Dec 1;17(12):e0277295. doi: 10.1371/journal.pone.0277295 (PMC9714722; doi:10.1371/journal.pone.0277295)
Supplement: S5 Appendix — Figure that shows two excerpts of actual conversations with the virtual coach. The excerpts include the last state question and persuasion based on the persuasion types authority and action planning. (PDF) [file pone.0277295.s005.pdf]

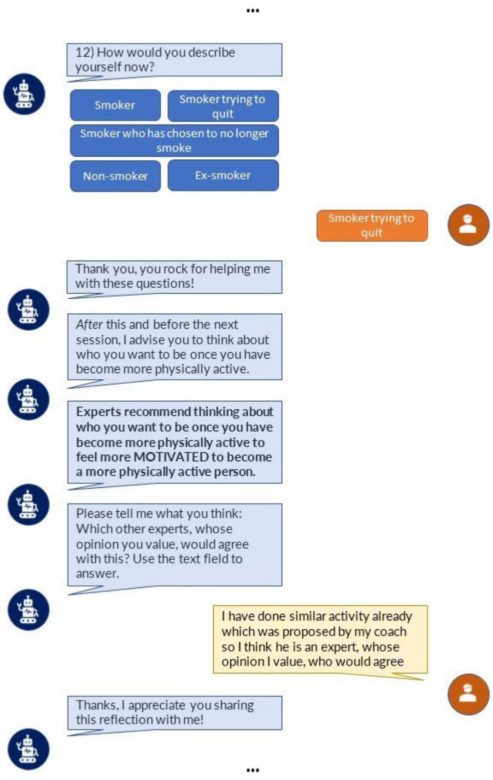

(a) Authority.

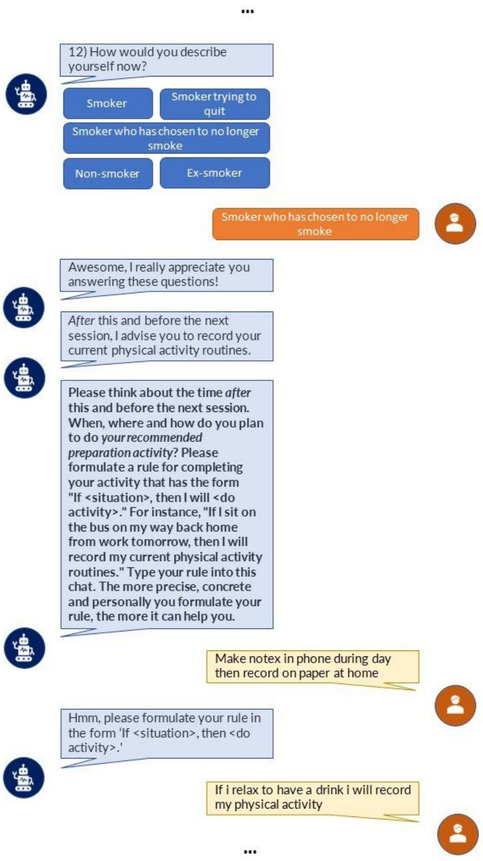

(b) Action planning.

Figure that shows two excerpts of actual conversations with the virtual coach. The excerpts include the last state question and persuasion based on the persuasion types authority (a) and action planning (b).
