# Supplementary material for: Addressing people’s current and future states in a reinforcement learning algorithm for persuading to quit smoking and to be physically active
Source: PLoS One. 2022 Dec 1;17(12):e0277295. doi: 10.1371/journal.pone.0277295 (PMC9714722; doi:10.1371/journal.pone.0277295)
Supplement: S6 Appendix — Table that shows the interpretation guidelines for Cohen’s κ based on Landis and Koch [93]. (PDF) [file pone.0277295.s006.pdf]

Table that shows the interpretation guidelines for Cohen's $\kappa$ based on Landis and Koch [49].

| **Cohen's $\kappa$** | **Evaluation** |
|---|---|
| $\leq 0$ | No agreement |
| 0.01-0.20 | No to slight agreement |
| 0.21-0.40 | Fair agreement |
| 0.41-0.60 | Moderate agreement |
| 0.61-0.80 | Substantial agreement |
| 0.81-1.00 | Almost perfect agreement |
