# Supplementary material for: Addressing people’s current and future states in a reinforcement learning algorithm for persuading to quit smoking and to be physically active
Source: PLoS One. 2022 Dec 1;17(12):e0277295. doi: 10.1371/journal.pone.0277295 (PMC9714722; doi:10.1371/journal.pone.0277295)
Supplement: S7 Appendix — Table that shows for each activity the number of times participants reported the effort they spent on the activity and the mean effort that was reported. We include only the samples used in our analysis for H1 (i.e., not the five samples of participants that were never assigned to a condition due to not completing session 2 and which were only used in the analysis for H2). (PDF) [file pone.0277295.s007.pdf]

Table that shows for each activity the number of times participants reported the effort they spent on the activity and the mean effort that was reported. We include only the samples used in our analysis for H1 (i.e., not the five samples of participants that were never assigned to a condition due to not completing session 2 and which were only used in the analysis for H2).

	**Activity**	**Number**	**Effort Mean (SD)**
SMOKING CESSATION			
1	Desired future self (writing)	114	5.91 (2.28)
2	Reasons for quitting smoking	131	5.65 (2.88)
3	Feared future self (writing)	82	5.15 (2.94)
4	Feared future self (picture)	96	5.75 (2.62)
5	Fighting match visualization	118	5.34 (2.70)
6	Smoking-relevant routines*	7	5.86 (3.24)
7	High risk situations*	7	7.71 (1.50)
8	Coping with cravings	127	5.87 (2.52)
9	Relaxation exercise	136	5.18 (3.16)
10	Personal rule	113	4.88 (3.09)
11	Education on body repair	103	5.22 (3.07)
12	Recording smoking behavior	140	5.69 (2.96)
**Total**		**1174**	**5.49 (2.85)**
PHYSICAL ACTIVITY INCREASE			
13	Recording physical activity	128	5.39 (2.73)
14	Barriers	136	5.60 (2.66)
15	Reasons for becoming more physically active	125	5.80 (2.56)
16	Recommended physical activity*	3	4.33 (4.04)
17	Desired future self (writing)	124	5.27 (2.47)
18	Feared future self (writing)	95	5.84 (2.76)
19	Feared future self (picture)	95	5.35 (2.81)
20	Fighting match visualization	127	5.00 (2.75)
21	Desired future self (picture)	119	4.76 (2.75)
22	Plan for becoming more physically active*	5	5.00 (2.65)
23	Impact of physical activity on cravings	121	5.18 (3.24)
24	Personal rule	109	5.21 (3.13)
**Total**		**1187**	**5.33 (2.80)**

Abbreviations: SD, Standard deviation.
* Activity had another activity as a prerequisite.
