# Supplementary material for: Addressing people’s current and future states in a reinforcement learning algorithm for persuading to quit smoking and to be physically active
Source: PLoS One. 2022 Dec 1;17(12):e0277295. doi: 10.1371/journal.pone.0277295 (PMC9714722; doi:10.1371/journal.pone.0277295)
Supplement: S9 Appendix — Figure that shows the mean L1-errors based on 100 repetitions when drawing different numbers of samples from the 2366 samples we gathered. We provide mean L1-errors for comparing the estimated and true Q-values for all state-action combinations (yellow) and comparing the true Q-values of the estimated and true optimal actions for all states (blue). True Q-values and optimal actions are those that are computed based on all 2366 samples. The horizontal lines indicate percentages of the mean L1-error for the lowest number of samples compared to the highest number of samples for comparing the estimated and true Q-values for all state-action combinations. (PDF) [file pone.0277295.s009.pdf]

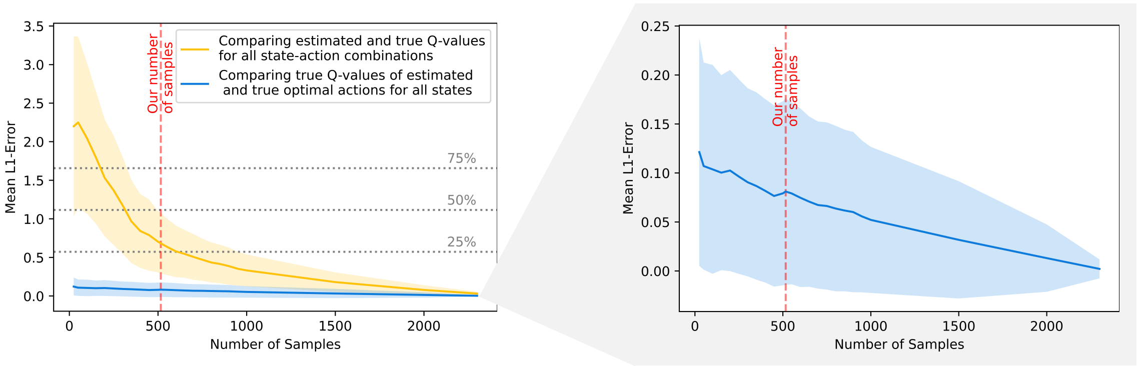

Figure that shows the mean L_1-errors based on 100 repetitions when drawing different numbers of samples from the 2366 samples we gathered. We provide mean L_1-errors for comparing the estimated and true Q-values for all state-action combinations (yellow) and comparing the true Q-values of the estimated and true optimal actions for all states (blue). True Q-values and optimal actions are those that are computed based on all 2366 samples. The horizontal lines indicate percentages of the mean L_1-error for the lowest number of samples compared to the highest number of samples for comparing the estimated and true Q-values for all state-action combinations.
