# Supplementary material for: Addressing people’s current and future states in a reinforcement learning algorithm for persuading to quit smoking and to be physically active
Source: PLoS One. 2022 Dec 1;17(12):e0277295. doi: 10.1371/journal.pone.0277295 (PMC9714722; doi:10.1371/journal.pone.0277295)
Supplement: Attachment — Submitted filename: Response to Reviewers.pdf [file pone.0277295.s010.pdf]

Dear Fu Lee Wang,

We would first like to express our gratitude for your efforts in helping us improve our manuscript. The remarks have been overall very helpful in pinpointing weaknesses and points of unclarity in the submission. We have addressed each reviewer comment to the best of our ability given the requirements of the journal. Below, we provide the details on how each specific comment was addressed. We further deliver the edited manuscript as well as a file that highlights the changes that we made to facilitate finding back the mentioned changes. We will refer to line numbers in the file with tracked changes while describing how we addressed the reviewer comments below.

Kind regards,

Nele Albers

Reviewer #1:

Comment 1.1: "*The paper describes research that aims to investigate whether a reinforcement learning approach (taking into account current and future states) can be used to determine suitable persuasion types in order to persuade people to do preparatory activities for smoking cessation. The conducted study is a longitudinal study through which they compared 4 increasingly more complex versions of their algorithm. The reasoning behind algorithm components incorporates elements of the behavioural change field (such as Com-B) with persuasive technology approaches (such as the PSD model), which places the study at an intersection of two highly relevant fields.*

The paper is clearly written and motivates well why certain factors are included and others are not. The steps taken are also relevant and well described. Furthermore, the data for this study is available at the indicated location and is neatly structured. The repository includes a README describing the contents, which also details how to perform the steps required to reproduce the data pre-processing and analysis. These steps are then further elaborated in similar README and manual files within the relevant subfolders. In addition, the authors also provide the code for the chatbot that was used in conducting the study.

All in all, the work appears to adhere to the 7 PLOS One criteria and investigates an important area of research since behaviour change applications still face major challenges with providing the right content at the right moment - e.g., leading to high dropout rates. Although the study's statistical analyses seem solid and clearly detailed, I must state that I'm not familiar enough with the specific Bayesian approach that was used to completely judge those steps thoroughly."

- ➔ We thank the reviewer for these evaluations of our manuscript, provided data and code, and relevance of our work.

Comment 1.2: "*The text and colored lines in almost all Figures 2-4 are quite blurry, the authors might want to increase the resolution for these. A minor note is also that the color coding for numbers in Figures 2 and 4 might not be suitable for people who suffer from colorblindness. However, in practice this is mainly a concern for the indication of sessions in Figure 4 since for Figure 2 the discussion in the main text provides sufficient context to not need the colors.*"

- ➔ Thanks for this note on colorblind-friendliness. We have checked and improved our color-coding in all our figures now (e.g., using this website: https://davidmathlogic.com/colorblind). Regarding the blurriness, we also noticed this in hindsight. We now increased the resolutions of Figures 2-5 as well as the figures in Appendix S2 and S4. Unfortunately, the Plos one figure processing tool made the figures slightly more blurry again, but we think that the figures are sufficiently clear now.

Comment 1.3: *"Line 12-13: "So Janine should also consider what type of person Janine is." -> 2nd Janine = Martha?"*

➔ Indeed, we have fixed this now to use "Martha" for the second occurrence of "Janine" (see line 13 in the manuscript with tracked changes).

Comment 1.4: *"Line 69-79: "[..] how thoroughly people [?] to their activities." -> missing verb?"*

➔ Thanks for noticing – there was a typo here. We now replaced "to" with "do". See line 81 in the manuscript with tracked changes.

Comment 1.5: *"Line 79: The 'cold start problem' comes to mind, you touch upon this in your discussion, but might be worth a short elaboration."*

➔ This is a good point. We had written that the simplest case would be to use some data on other people, but of course such data may not exist at the start. We have now added that in case of no available data on other people, the virtual coach would need to choose either randomly or based on other sources of information such as experts. See line 94 and the corresponding footnote in the manuscript with tracked changes.

Comment 1.6: *"Line 614: Perhaps add 'in the data repository'?"*

➔ We have now rephrased this part to make clear that we talk about the published data. If our manuscript is accepted and we publish our data, we will also add the reference to the data there again. See line 716 in the manuscript with tracked changes.

Comment 1.7: *"Finally, one minor comment regarding contents of the data repository: The text in the codeblocks of a number of pdf files detailing how to perform the analyses runs outside of the codeblocks and often also the pages. E.g., in the Bayesian Comparison pdf file and the pdf files for the 1st Hypotheses."*

➔ Thanks for pointing this out. We have now re-knitted Rmd-files using the package "formatR" that allows us to wrap lines.

Reviewer #2:

Comment 2.1: *"While, in the submitted version of the article, the authors show substantial and impressive knowledge about the foundations and methods of social psychology (far beyond the assessment capabilities of this reviewer, to be honest) the account given of what they call 'algorithms' is definitely sketchy, to say the least, and insufficient to evaluate how the experiments were performed. More precisely, to this reviewer, the section 'Methods', as it stands, does not give an adequate description of the algorithmic technique(s) that the authors adopted in the study.*

Below, I try to convey some more specific comments and suggestions for improvement that, to this reviewer, should be introduced in the due course of a major revision."

- ➔ We thank the reviewer for the evaluation of the manuscript's content with regards to foundations and methods of social psychology. We also very much value the reviewer's critical feedback on the description of our algorithmic approach. Below, we show how we have now addressed each of the reviewer's concerns.

Complexity

Comment 2.2: *"In theoretical computer science, the term 'algorithm complexity' designates very precise measurements: e.g, time complexity, memory complexity. In contrast, the authors use this term in a rather qualitative way, which I think should be better explained. The description of the so-called four levels of complexity of the algorithms are only accessible to the reader in Figure 2 and never explained in a satisfactory way. For clarity, this reviewer thinks that they should be summarized in a table, possibly within the main text and not in the appendix, and a precise methodological description should be given for each level."*

- ➔ We agree with the reviewer that in light of the central character if these algorithm complexity levels to our study, they should be more thoroughly explained. To our in-text description of the four algorithm complexity levels in the section "Experimental design", we have now added a table that lists for each algorithm complexity level how a persuasion type is chosen and which policy we obtained after training the algorithm complexity level on the data from the first batch of people (N = 516) who successfully completed session 2 (now Table 1). We also refer back to this table as we describe the algorithm components (e.g., state space, action space) in the "Algorithm"-section of the manuscript (e.g., see line 330 in the manuscript with tracked changes).

Algorithm

Comment 2.3: *"In the section 'Methods' and in the sub-section 'Algorithm' the authors characterize the overall RL framework in terms of a Markov Decision Process. Along this line, they describe the Action Space, State Space and Reward in a certain length (no objections here). What looks to be completely missing from that point on, however, is a description of the actual RL approach adopted. Below a list of a few remarkable doubts that remain unsolved:"*

- ➔ We are happy to hear that the description of the action space, state space, and reward is satisfactory. We will describe below how we address each of the reviewer's concerns with regards to the description of the adopted RL approach.

Comment 2.4: *"The all-important MDP Transition Function is just mentioned but not described. The reader may conjecture that the authors implied that they could not know the MDP Transition Function beforehand, which would be reasonable, given the novelty of the approach. Is this the case?"*

- ➔ This is a good point. We have now added a section called "Reward and transition functions" that describes that the reward and transition functions are not known beforehand but estimated from the samples gathered in sessions 1 and 2 from the first batch of people (N = 516) who successfully completed session 2. See lines 299 to 302 in the manuscript with tracked changes.

Comment 2.5: *"In 'complexity levels' 3 and 4 (see Figure 2) the authors hint at the Q-value of each action, given a specific state. According to basic RL textbooks [1], the Q-value can be either computed beforehand, if the transition function is known in full, or learned from experience, like for instance with Q-learning, otherwise. Which approach is the one taken by the authors is unclear."*

- ➔ As described for Comment 2.4, the transition function was not known beforehand and instead estimated based on the samples gathered in sessions 1 and 2 from the first batch of people (N = 516) who successfully completed session 2. We have now added to the new "Algorithm training"-section of the manuscript that the Q-values were computed based on the estimated reward and transition functions via value iteration. We have now also added that no samples were later added to the training set of 516 samples that the reward and transition function estimates were based on so that the policies for all people were trained on the same number of samples and hence comparable. Please refer lines 327 to 334 in the manuscript with tracked changes. Furthermore, we have now formally defined the Q-value function at the start of the "Algorithm"-section. See lines 227 to 229 in the manuscript with tracked changes.

Comment 2.6: *"3.1) In RL the policy is the objective of learning. 'One policy, multiple subjects' could be a fitting description, by default. Now if the policy is per-subject, as the above text seems to imply, how is such policy learned? Typical RL optimization processes require a large number of episodes, not just one interaction, however protracted over time."*

- ➔ This comment from the reviewer shows that it was not sufficiently clear how the policies for algorithm complexity level 4 were computed. We have now improved the section on "Similarity computation" to make this clearer. Specifically, the same 516 training samples used for the other algorithm complexity levels are also used for level 4. The difference is in how these samples are weighted when the individual policies pi_i are computed. More precisely, a sample from person j when computing the policy for person i is weighted based on how similar i and j are. A larger weight is thereby given to samples from more similar people. These weights are considered when estimating the individual reward and transition functions for person i, which form the basis of the individual Q-values for person i, based on which pi_i is calculated. Equation 2 now shows how the sample weights are incorporated into the estimation of the reward function for person i. Please refer to lines 323 to 326 in the manuscript with tracked changes.

➔ An alternative to weighting samples based on similarity is to use only samples from an individual person. We proposed looking into this as part of our discussion, which we have now reformulated to make this idea clearer. Of course, more interactions with an individual would be needed in such a setting. Please refer to lines 775 to 778 in the manuscript with tracked changes.

<u>Comment 2.7</u>: *“3.2) What is precisely a ‘sample’, to the authors? Is that the typical tuple used in RL? And if so, with what method is policy \pi_i updated?”*

➔ This is very helpful point. We realized that it would be helpful to use the notation <s_i, a_i, r_i, s’_i> for the samples to make clear that we keep track of the people corresponding to the samples for algorithm complexity level 4. We have used this notation in the part we added to the “Similarity computation”-section as part of Comment 2.6. See line 325 in the manuscript with tracked changes.

➔ We also noticed that the formulation of “updating” pi_i in the “Similarity computation”-section is confusing, because pi_i is computed only once for each person in our study based on the 516 training samples used for all algorithm complexity levels. We have replaced the word “updating” with the word “computing” to make this clearer (see line 305 in the manuscript with tracked changes). For how pi_i is computed, please refer to Comment 2.6.

<u>Comment 2.8</u>: *“3.3) If, in ‘complexity level’ 4, samples are collected not only for individual i but for a cohort of other individuals j, apart from the similarity measure (whose definition is given in the article), how is such evidence combined to have a policy in the sense of RL?”*

➔ As described for Comment 2.6, all samples are weighted based on the similarity of the corresponding people j to the person i we want to compute a policy pi_i for. These weights are used to estimate individual reward and transition functions, which are used to compute individual Q-values. These individual Q-values are used to compute individual policies pi_i. We have now added Equation 2 to show how the sample weights are used to compute an individual reward function. We have added these explanations in the section “Similarity computation.” See lines 323 to 326 in the manuscript with tracked changes.

<u>Comment 2.9</u>: *“3.4) If samples from other individuals are used to update individual’s policy, how is time synchronization handled? In other terms, give that RL is incremental, subjects participating the experiments at later times may benefit from a larger quantity of samples collected from other subjects.”*

➔ This comment shows that it was not clear that the policies for all four algorithm complexity levels were only computed once based on the training set of 516 samples mentioned previously to make sure that all policies were trained on the same number of samples. We have added this to the new “Algorithm training”-section as part of addressing the reviewer’s Comment 2.5 (see lines 327 to 334 in the manuscript with tracked changes), as well as to the caption of Table 1 that we added

for Comment 2.2. In addition, as we have described in addressing the reviewer's Comment 2.7, we have replaced the word "updating" at the start of the "Similarity computation"-section with "computing" when talking about pi_i to make this clearer (see line 305 in the manuscript with tracked changes).

Statistics

Comment 2.10: *"Once again the focus is on the algorithmic part. In fact, 'complexity level' 1 and 2 imply knowing the 'best average reward' and 'best average reward in state', respectively. How are these values computed? Are they determined during the random persuasion stages?"*

- ➔ Indeed, the "best average reward" and "best average reward in state" for algorithm complexity levels 1 and 2 are also computed based on the training set of 516 samples from the first batch of people who successfully completed session 2. We have addressed this as part of Comment 2.5, where we made this clearer in the new "Algorithm training"-section of the paper. Please refer to lines 327 to 334 in the manuscript with tracked changes. The formal definitions of algorithm complexity levels 1 and 2 are shown in the new Table 1 that we added as part of the reviewer's Comment 2.2.

Abstract

Comment 2.11: *"To test the added value of subsequently incorporating these elements'*
In the previous sentence we read "... variables such as ..." followed by some examples, but there is no specific term stating explicitly which elements the authors are talking about (Psychological dimensions? Past experiences?)"

- ➔ This helpful comment from the reviewer shows that the use of the terms "variables", "aspects" and "elements" in the abstract was confusing. The word "variables" only referred to information about the user's current state (e.g., self-efficacy). We have now changed the wording in the abstract to make this clearer.

Comment 2.12: *"'training the algorithm separately for the two types of activities may be more effective than training one single algorithm on the combined data set.' Is it or is it not more effective? This is crucial to establish the contribution of the article."*

- ➔ This is a good point. We have now rephrased this part of the abstract to make clearer what our findings for the second hypothesis are. More precisely, we found low agreement between the optimal policies computed separately for the two types of activities, suggesting limited policy generalizability between the two types of activities.

Text

Comment 2.13: *"Line 13: Possible typo ' Martha'"*

➔ Thanks for noticing this. We have changed “Janine” to “Martha” as part of Comment 1.3 from the first reviewer. See line 13 in the manuscript with tracked changes.

Comment 2.14: *“Line 73+: Such statement would deserve some reference in literature”*

➔ This comment shows that it was not clear that this statement was a preview of our second hypothesis, which is justified later in the section. We have now rephrased the statement to make this clearer. See lines 85 to 87 in the manuscript with tracked changes.

Comment 2.15: *“Line 202+: The level of precision in defining the discount factor is somewhat surprising, given the overall account of the algorithm (see also above)”*

➔ We have now extended our description of the other algorithm parts based on the reviewer’s earlier comments to match the level of detail used to describe the discount factor. For example, see the new section called “Reward and transition functions” in lines 299 to 302 in the manuscript with tracked changes.

Comment 2.16: “*Line 264: Have the authors considered measures other that the Euclidean*?”

➔ We have used only the Euclidean distance as it is a commonly used distance. We have now added the use of other distances as a direction for future work to the “Discussion”-section of the manuscript based on the reviewer’s comment. Please refer to lines 717 to 719 in the manuscript with tracked changes.

Comment 2.17: *“Line 435: How have entry errors been corrected?”*

➔ This comment shows that this part about correcting entry errors needed to be better explained. We have now added more details to the “Data preparation and analysis strategies”-section. Specifically, we corrected a total of 4 entry errors that people messaged us about in Prolific right after their sessions. In two of these cases, these were attention check questions, whose answers influence neither the conversation nor the persuasion in any way. In the other two cases, participants corrected their answers to the state question about smoker identity in session 1. As people were persuaded randomly in the first session, these entry errors also did not influence the conversation or persuasion in any way. Corrections could be made because the policies were not trained online. Instead, they were computed once on a fixed training set of 516 samples from session 1 and 2 (see our responses to the reviewer’s earlier comments). Please refer to lines 518 to 524 in the manuscript with tracked changes.

Comment 2.18: *“Table 4: Being the mean perceived motivational impact computed on values which go from -10 to 10, the improvements obtained in this table are very low (The min and max values differ of 5.40 - 4.64 = 0.76, which represents a maximum improvement of 0.76/21 = 3.3%): is this correct? Such result should be further described (and better discussed).”*

- ➔ In light of this comment from the reviewer, we have now added a few sentences at the start of the "Results"-section that give an intuition for the size of the observed effects for effort and perceived motivational impact (see lines 610 to 615 in the manuscript with tracked changes). We had already discussed the effects as small for effort in the "Discussion"-section, and have now added a discussion of the small effects for the perceived motivational impact there as well. Please refer to lines 793 to 799 in the manuscript with tracked changes.

Comment 2.19: *"Line 595: By looking at the measured moments in Tab.3, the statement doesn't seem evident. It could be useful to have a graph showing the evolution of the measures according to the moments."*

- ➔ This comment from the reviewer shows that it was not sufficiently clear how we came to this conclusion. We now refer to the three-way interaction effect as the basis for this conclusion, and also refer to the increase in effort between session 3 and 4 for algorithm complexity level 3 shown in Figure 4A. Please refer to lines 696 to 698 in the manuscript with tracked changes.

Reviewer #3:

Comment 3.1: *"This is a well-written paper with thorough description and evidence provided in attachments and a link where all the data and scripts are available. The authors apply Reinforcement Learning techniques to behavior change problems and there are still a lot of interesting challenges to tackle in this domain. The authors collected new data to examine whether different algorithm complexities would contribute to better performance of a reinforcement learning agent that was delivering persuasive messages to support people to change their physical activity or smoking behavior. The authors did a commendable job in providing clear stats of demographics within groups and to provide clear descriptions of their data and model in the readme files.*
The research is novel and of interest, although the outcomes give us only limited insights."

- ➔ We thank the reviewer for this positive evaluation of our work with regards to the writing itself, provision of description and evidence in attachments, description of the data, model, and demographics within groups, and novelty.

Major comments

Comment 3.2: "*1. The research is based on two type of user behaviors: physical activity and smoking cessattion behavior. It was assumed that people could be persuaded in similar ways for those activities because 'they serve the same behavioral goal: quitting smoking.' This is a major assumption underlying the entire research and it seems flimsy and not sufficient. There are many behaviors that are very different but could contribute to the same goal. Moreover, smoking is addictive and as such I would expect at least that this would be taken into account for any persuasive strategy.*"

- ➔ This comment from the reviewer shows that it was not sufficiently clear what the activities were that people were persuaded to do. Specifically, rather than persuading people to quit smoking or become more physically active, the virtual coach persuaded people to *prepare* for doing so. Such preparatory activities included writing down reasons for wanting to quit smoking, writing down reasons for wanting to become more physically active, or tracking one's smoking or physical activity behavior. We have taken two steps to make this clearer. First, we have now adapted the description of the preparatory activities from the "Materials"-section of the paper: we have now included the text for one activity in the main part of the paper (see lines 383 to 386 in the manuscript with tracked changes). The remaining activity formulations can be found in Appendix S8. Second, we have clarified the types of activities used at the end of the "Introduction"-section. Please refer to lines 65 to 69 as well as line 73 in the manuscript with tracked changes.

<u>Comment 3.3</u>: *"2. Algorithm description: the paper would benefit from a formal definition of all components of the algorithm, like action space and state space."*

- ➔ In light of this helpful comment, we have now added to our in-text description of the algorithm components in the "Algorithm"-section Table 2 that provides a concise and formal overview of them.

<u>Comment 3.4</u>: *"3. The persuasive messages seem not very personalized and quite general ('experts', 'people like you'). I would think it difficult to achieve true engagement from them. I do appreciate that the authors looked at other literautre to find templates that were validated, but would be interested in their opinion of how the content of their messages might have influenced the result."*

- ➔ We agree with the reviewer that there may be a limited effect of such persuasive messages by themselves. We tried to address this in our study by also including reflective questions that asked people to reflect on the persuasive message they received (see Table 3), as well as by including a reminder question in the reminder messages people received in Prolific after the session (see Table 4). These questions were meant to increase self-referencing, which can increase the in-depth processing of messages according to the elaboration likelihood model. Nevertheless, our results show that the effect of persuasive messages is small. This is in line with previous work using other persuasive messages, as we discuss in the "Discussion"-section. However, the reviewer is right in that we as a community may have missed more effective forms of persuasive messages. We have now added this to the "Discussion"-section. Please refer to line 799 in the manuscript with tracked changes.

<u>Comment 3.5</u>: *"4. The authors have only included self-report of motivation and effort for behavior as a measure of behavior change. This seems quite limited, in particular because the self-report was only for preperatory activities and not the actual behavior itself (smoking). Please include more information on what those type of activities are. It seems a limitation that*

the study does not evaluate whether the actual goal/target behavior was affected and the paper needs more argumentation on why this approach was chosen and even appropriate."

- ➔ Similarly to Comment 3.2, this comment from the reviewer shows the lack of clarity regarding the nature of the activities people were persuaded to do. Specifically, the activities only related to *preparing* for quitting smoking or becoming more physically active. Activities included things such as writing down reasons for quitting smoking, tracking one's physical activity behavior, or thinking about barriers for becoming more physically active. Participants were not asked to change their smoking or physical activity behavior in any way. As described for Comment 3.2, we have addressed this by 1) explaining in more detail in the "Materials"-section what the activities were, including a formulation of one activity in the main text and a reference to the other activities in Appendix S8, and 2) explaining also at the end of the 'Introduction'-section in more detail what the intervention looked like.
- ➔ Moreover, this comment shows that our approach of testing primarily a persuasion algorithm rather than a full behavior change intervention required more explanation and justification. We have now added this to the end of the "Introduction"-section. Specifically, since the effects of the persuasion algorithm components are difficult to assess in a complete behavior change intervention in which many other components such as goal-setting and progress feedback can play a role, we created a minimal intervention in which people were only coached to *prepare* for changing their behavior. Please refer to lines 65 to 69 in the manuscript with tracked changes.
- ➔ In addition, the reviewer points out that our study is based on self-reported measures of behavior and predictors of behavior (i.e., the effort people spent on their preparatory activities and the perceived motivational impact of the conversational sessions). We have now added to the "Discussion"-section that the use of more objective measures of behavior would be an interesting direction for future work. See lines 773 to 775 in the manuscript with tracked changes.

<u>Comment 3.6</u>: "*5. It seems the algorithm had very limited data to train on, given that it was on the data of just 2 sessions. So it has very limited information on which action would yield the best reward. Also, the study uses results of 521 participants, while 528 was required (and a 'conservative' number at that). In limitations is should be adressed how this could impact the conclusions and follow-up."*

- ➔ First, the reviewer's comment shows that it was not sufficiently clear that we had less than 528 participants only for the last measurement moment (see S2 Appendix) and were able to use data on more participants than 528 for the other measurement moments due to the hierarchical nature of our statistical models. These models allow for using partial data on participants. We now tried to make this clearer. See line 474 in the manuscript with tracked changes.
- ➔ Second, our a priori sample size estimation resulted in a value of 528 based on a binary dependent variable. Since we used interval rather than binary dependent

variables, this estimate was conservative, as interval dependent variables need no more samples than binary ones. Nevertheless, the reviewer is right in that we did not reach our a priori sample size. We thus now added the results of a Bayesian power analysis for 129 samples per condition, which is the lowest number of samples we obtained for a measurement moment for a condition. This resulted in a power of 0.78. See lines 460 to 467 in the manuscript with tracked changes. We also now mention this power in the “Discussion”-section. Please refer to lines 809 to 810 in the manuscript with tracked changes.

➔ Third, we have now added our rationale for the number of samples used for training the algorithm complexity levels. More precisely, our choice of the number of samples was based on the guidelines for multiple regression analysis for a medium effect, an alpha of 0.05 and 3 independent variables (i.e., the 3 state features). This requires a sample size of 76 according to Cohen. Since we had 5 possible actions, we multiplied this by 5 for a value of 380. We have now added this reasoning to the new “Algorithm training”-section of the manuscript. See lines 335 to 341 in the manuscript with tracked changes.

➔ Fourth, to see how well the 516 training samples allow us to predict Q-values and optimal actions, we have now added an analysis of the impact of different sample sizes. Please refer to lines 341 to 352 in the manuscript with tracked changes, as well as S9 Appendix.

<u>Comment 3.7</u>: “*6. Please include results of how the agent was doing in terms of reward for the different groups and how this relates to the (involvement in) behavior that was done.*”

➔ This comment from the reviewer indicates that it was not clear that the effort per session and algorithm complexity level shown in Figure 4A gives an indication of the reward obtained by the agent, as the reward is computed based on the effort. We have now added this clarification to the beginning of the “Results”-section. Figure 4B and 4C also show the effort per session and algorithm complexity level for people with low and high involvement in their activities. Please refer to lines 607 to 609 in the manuscript with tracked changes.

Minor comments:

<u>Comment 3.8:</u> “*1. The authors seem to generalize results to all behavior change applications from the start, where they assert that all applications assign their users activities. This seems a bold statement and does not link to any definition of behavior change or e-coaching solutions.*”.

➔ This is a good point. We have now added the word “often” to make clear that several but not all behavior change applications assign their users activities.

<u>Comment 3.9</u>: “*2. How do the interventions link to habit formation? Doing a certain prep behavior once or repeatedly should have different value.*”

- ➔ This comment from the reviewer points out that we did not make clear that people were always assigned a different preparatory activity so that the activities never repeated for a person. So the goal was not to create a habit for doing a certain preparatory activity. We have now made that clearer in the "Materials"-section of the manuscript. See lines 401 to 402 in the manuscript with tracked changes.

Comment 3.10: "*3. It is unclear to what extent people were asked to do activities that they would normally not do, or that would be new to them. Also, did people that were included try to stop smoking before? This might impact their behavior.*"

- ➔ We think this comment from the reviewer is connected to Comment 3.9 and Comment 3.2 in that it was not sufficiently clear what the activities were and that each person was always assigned a new activity in each session so that the activities never repeated for a person. Please refer to Comment 3.9 and Comment 3.2 for how we addressed these issues.
- ➔ In addition, the reviewer indicated that it would be good to know whether people had previously tried to quit smoking. We have therefore now added to the table in Appendix S3 with participant characteristics whether participants indicated having previously tried to quit smoking for at least 24 hours. We also now mention in the "Participants"-section that this information is available in Appendix S3 (see line 486 in the manuscript with tracked changes). Notably, however, people were only eligible for our study if they were not currently part of another intervention to quit smoking, as described in the "Participants"-section of the paper.

Comment 3.11: "*4. Please include to what extent the message 'journey' indeed differed for participants in complexity 1-4.*"

- ➔ This is a good point. In combination with Comment 2.2 from the second reviewer, we have now created a new table (Table 1) that lists for each algorithm complexity level the way of choosing persuasion types and the policy that was learned.

Comment 3.12: "*5. Image 1 should be improved, it is unclear whether the actor is the human or the agent. Table 3 and 4 need more axes descriptions (not 1,2,3,4) and scale.*"

- ➔ We thank the reviewer for noticing this. We have now added symbols for the actor in Figure 1 (i.e., virtual coach vs. human). In addition, we have now added the names of the algorithm complexity levels in these two tables, which are now called Table 5 and Table 6 due to the addition of two tables to the paper. We also mention below the tables the scale that the respective measure was measured on.

Besides addressing these helpful comments from the reviewers, we have also further improved our work by 1) improving the language, 2) adding a few references, 3) updating the Dockerfile provided with the code to newer package versions, 4) rerunning all analyses in the updated Docker containers, resulting in slightly different values for some values from the Bayesian analyses, and 5) fixing an error in our preprocessing code that resulted in two out of the more than 2300 samples being wrongly removed for failing attention checks for the analysis for hypothesis H1. We have rerun our analysis thereafter and now report the corrected results.
