# Supplementary material for: Addressing people’s current and future states in a reinforcement learning algorithm for persuading to quit smoking and to be physically active
Source: PLoS One. 2022 Dec 1;17(12):e0277295. doi: 10.1371/journal.pone.0277295 (PMC9714722; doi:10.1371/journal.pone.0277295)
Supplement: Attachment — Submitted filename: Response to Reviewers.pdf [file pone.0277295.s011.pdf]

Dear Fu Lee Wang,

Thank you for this second round of examining our manuscript and providing recommendations for improving it. We were happy to see that we succeeded in addressing many of our reviewers' comments in our first revision. The new remarks from the reviewers have overall helped to identify further points of unclarity in our submission. In this second revision of our article, we have addressed each new reviewer comment to the best of our ability given the requirements of the journal and the comments from the first review round. Below, we provide the details on how each specific comment was addressed. We further deliver the edited manuscript as well as a file that highlights the changes that we made to facilitate finding back the mentioned changes. We will refer to line numbers in the file with tracked changes while describing how we addressed the reviewer comments below.

Kind regards,

Nele Albers

Reviewer #1:

Comment 1.1: *"Thank you for the substantial revisions that have been made, not just regarding my comments and suggestions, but also regarding those of the other reviewers. The revision has addressed al my aforementioned comments. I've also reviewed the authors' responses to the other two reviewers and they seem to have made changes addressing their comments thoroughly as well, although I am not an expert on the specific type of algorithm employed by the authors. I therefore leave the final judgement on the changes regarding the other reviewers' questions to those respective reviewers."*

- ➔ We are happy to hear that we have successfully addressed all comments from this reviewer.

Reviewer #2:

Comment 2.1: *"To this reviewer, revision R1 shows substantial progresses in the direction requested. In particular, the fundamental algorithmic aspects of the approach proposed are described in a rather satisfactory way and the overall method can now be evaluated in full. As a word of caution, however, the authors should be aware that many of the algorithmic ideas that they adopt, Reinforcement Learning in the first place, are elaborated in a very specific way, to adapt these to the authors' objectives. This is not a limitation 'per se' but calls for extra care in the description of the method proposed, to avoid generating confusion with readers being acquainted with more (allow me) 'canonical' versions of the techniques in point."*

- ➔ It is good to see that we have made progress in describing our algorithmic approach in the eyes of the reviewer. We also appreciate the recommendation to be careful in how our algorithmic approach is understood by people who are more familiar with more standard versions of reinforcement learning. We agree that it is a challenge to describe the algorithmic approach, especially since potential readers of this paper include both psychologists who are not at all familiar with reinforcement learning and people who are very familiar with a more standard application of reinforcement learning.

Comment 2.2: *"Table 1 is a very welcome enhancement to the article, however, is literally plenty of aspects that cannot be understood in a first reading, since they require integrating other aspects that are clarified much further on. Also Table 2 is very useful, although it could be improved further."*

- ➔ We are glad to see that Table 1 and Table 2 are overall seen positively by the reviewer. We describe how we address the points for improvement suggested by the reviewer below.

Comment 2.3: "*put Table 2 ahead of Table 1 and modify the description of the algorithm in an incremental fashion"*

- ➔ We agree with the reviewer that some of the content of Table 1 is only clarified later on. In light of this comment from the reviewer as well as Comment 2.9, we have now broken up Table 1 into two tables. The first table summarizes the four algorithm complexity levels when they are first introduced as part of the "Experimental design"-section at the beginning of the "Methods"-section. Abbreviations used in this table are defined below the table. Moreover, when people are referred to Table 1 from the text as part of the "Experimental design"-section, they are informed that the algorithm components will be explained in the next section. The second table (now Table 5) now describes the learned policies for the four algorithm complexity levels as part of the "Algorithm training"-section. This means that people will now only see the learned policies from Table 5 once they have read the description of the algorithm components, including their summary in Table 2.

<u>Comment 2.4</u>: "*in Table 2 (will be Table 1) clarify how state space is defined, redirecting to text for better clarification*"

- ➔ We agree with the reviewer that the state space definition in Table 2 is alone not sufficient. We now follow the reviewer's suggestion and refer to the "State space"-section for further details from Table 2.

<u>Comment 2.5</u>: *"in Table 2 add the abbreviations for the Action Space, which will be used in Table 1 (will be Table 2)"*

- ➔ This is a good point – it would help for the terminology to be consistent between the tables. We had already defined the abbreviations below Table 1 (now Table 5). What we have now added is to use these abbreviations also in Table 2.

<u>Comment 2.6</u>: *"in Table 2, both reward function and transition function are just defined as function types, which is not very informative: at least a minimal description should be given"*

- ➔ In light of this comment, we have now added minimal descriptions for both the reward and the transition function to Table 2.

<u>Comment 2.7</u>: *"Table 1 contains a lengthy and somehow confusing repeated definition of how states are defined: it would be advisable to extract the definition of states, possibly by making it a separate and smaller table, and then quoting just states as binary combinations in Table 1"*

- ➔ Our goal is that both computer scientists and people without a background in computer science can understand the table. Specifically, people without a background in computer science may not be familiar with binary notation. We therefore chose to use this more elaborate description of what the states are. Moreover, the selected state features also differ between some of the conditions, so we think that it would be cumbersome to have to refer to another table to remember what the state features for a condition were. To make clearer that the features differ between the conditions, we have now added the feature number in parentheses in

what is now Table 5. Since this comment from the reviewer suggested that the table could be improved, we have now also asked several people for feedback. What they remarked is that it was not clear that the columns in the sub-tables correspond to states. We have thus now added numbers for the eight states in these sub-tables in what is now Table 5. We hope that this makes Table 5 easier to understand for people with and without backgrounds in computer science.

Comment 2.8: *"in fact, the fundamental information content of Table 1 is the relation between states (whenever this applies) and the most effective actions, according to the metrics of choice."*

➔ As described for Comment 2.3, we have now broken up Table 1 into two tables (Table 1 and Table 5) to separate the two types of information from the former Table 1. The first type of information is the definition of the four algorithm complexity levels as part of the "Experimental design"-section that first introduces the four levels (now Table 1). The second type of information is what the learned policies in the four algorithm complexity levels were as part of the "Algorithm training"-section (now Table 5).

Comment 2.9: *"In the light of the comments above, the authors should consider that the contents of Table 1 seem to be pertaining more to a 'Results' section rather to a 'Method' one. Therefore, some re-arrangement of the overall description should be performed, to reflect this."*

➔ We agree with this. As described for Comment 2.3, Table 1 has now been broken up into two tables, now Table 1 and Table 5. Table 5 now contains the learned policies and is referred to as part of the "Algorithm training"-section.

Comment 2.10: *"The cited paper*
Di Massimo F, Carfora V, Catellani P, Piastra M. Applying Psychology of Persuasion to Conversational Agents through Reinforcement Learning: an Exploratory Study. In: CEUR – Workshop Proceedings. vol. 2481; 2019.
has a journal version which I think should be cited in its place:
Carfora, V., Di Massimo, F., Rastelli, R., Catellani, P., & Piastra, M.. Dialogue management in conversational agents through psychology of persuasion and machine learning. Multimedia Tools and Applications, 39, 35949-35971; 2020."

➔ Thanks for pointing this out. We have changed this now.

Reviewer #3:

Comment 3.1: *"The authors adressed the majority of my comments well."*

➔ We are glad to hear this.

Comment 3.2: "*I would like to make to final remark for the authors to consider. It relates to my earlier comment (3.5), which is in essence about what constitutes success for this*

intervention. If you do an intervention which targets preperation for behavior, that preparation also contains behaviors that could be monitored (in this case for example watching a video and writing things down). To the reader it is not yet clear why self-report of effort is reliable as a (only) measure in this context (for instance, succesfull preparation could (and should) also translate to increased awareness or motivation, which was not assessed). Also, it would be nice to know how the authors aimed to make the self-report a more robust measure by mitigating reporting biases."

➔ We see two important points in this helpful comment from the reviewer. First, the question regarding how reporting biases for the self-reported effort were addressed. We had already mentioned in the "Participants"-section that loss aversion and self-interest biases were addressed by telling people that their effort responses would not affect their payment in any way. What we have now changed is to define in the "Participants"-section what loss aversion and self-interest biases are to make this clearer (see lines 467 to 470 in the document with marked changes). Second, the reviewer again raises a question regarding the suitability of the self-reported effort as reward signal. The choice of reward signal is indeed a very interesting and important one, which is why we have now created a new paragraph in the "Discussion"-section to expand on this. Let me briefly summarize here what we added to the paper. More precisely, we can think of the following chain of outcome measures: effort spent on activities -> "preparedness" for quitting smoking -> successful quitting and maintenance of smoking abstinence. While the more distal outcome measures describe the behaviors we actually want to see, using them as reward signal for comparing the four algorithm complexity levels leads to several challenges. This includes the time until we can measure the reward signal (e.g., several months or years to judge successful abstinence maintenance), the noisiness of the signal (e.g., a cancer diagnosis a year from now can affect abstinence maintenance positively), and the way of measuring the reward signal (e.g., since the preparatory activities are meant to do different things such as removing smoking cues or increasing self-efficacy, several questions would need to be asked, thus increasing the burden on users). Since the links in the chain from effort to successful maintenance of smoking abstinence have been supported by other work, we thus chose the effort as reward signal. What we have also added to the paper is that future work could look at more objective measures of behavior instead of the effort. This may, however, be challenging for some parts of activities, such as posting a rule for not smoking somewhere one can see it every day. See lines 745 to 772 in the document with tracked changes.

Additional Changes

In addition to addressing the comments from the reviewers, we have corrected some spelling errors, added a few more relevant references, checked references for retracted or updated papers, and updated the fonts in the figures. Moreover, since we have one additional table due to splitting up Table 1 based on the comments from the second reviewer, we also had to re-knit the statistical analysis pdf-files for the Bayesian analyses to contain the correct table numbers when referring to the paper.
